# Understanding Soaring Coronavirus Cases and the Effect of Contagion Policies in the UK

**DOI:** 10.3390/vaccines9070735

**Published:** 2021-07-03

**Authors:** Miguel A. Durán-Olivencia, Serafim Kalliadasis

**Affiliations:** Department of Chemical Engineering, Imperial College London, London SW7 2AZ, UK; m.duran-olivencia@imperial.ac.uk

**Keywords:** COVID-19, SARS-CoV-2, SIR

## Abstract

The number of new daily SARS-CoV-2 infections experienced an abrupt increase during the last quarter of 2020 in almost every European country. The phenomenological explanation offered was a new mutation of the virus, first identified in the UK. We use publicly available data in combination with a time-delayed controlled SIR model, which captures the effects of preventive measures on the spreading of the virus. We are able to reproduce the waves of infection occurred in the UK with a unique transmission rate, suggesting that the new SARS-CoV-2 variant is as transmissible as previous strains. Our findings indicate that the sudden surge in cases was, in fact, related to the relaxation of preventive measures and social awareness. We also simulate the combined effects of restrictions and vaccination campaigns in 2021, demonstrating that lockdown policies are not fully effective to flatten the curve. For effective mitigation, it is critical that the public keeps on high alert until vaccination reaches a critical threshold.

## 1. Introduction

**Figure 1:**
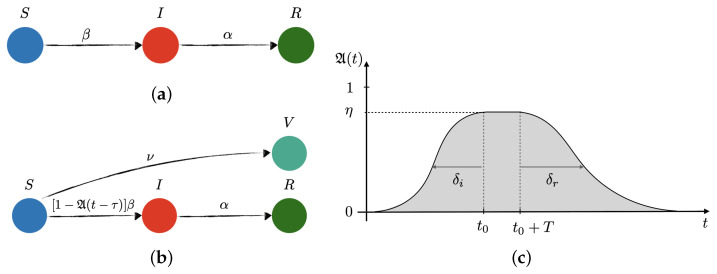
Sketch of transitions in the (**a**) free and (**b**) controlled SIR network models of disease transmission, and (**c**) model of the preventive social response, A(t). *S*, *I*, *R* and *V* stand for susceptible, infected, recovered, and vaccinated, respectively. Additionally, β, α and ν are the infection, recovery, and vaccination rates, respectively. In the controlled SIR model, the infection rate is reduced by the factor (1−A(t−τ)), which depends on the incubation time period, τ, the effectiveness of the social response, η∈[0,1], the time when the awareness reaches its peak, $t_{0}$, the time extension of the maximum level of alert, *T*, and the characteristic times for reaching maximum awareness, so-called social inertia, $\delta_{i}$, and the relaxation time after lifting restrictions, $\delta_{r}$, respectively.

The susceptible-infectious-recovered (SIR) model [1] (Figure 1a), is a popular mathematical model for computational scrutiny used in numerous studies, often to estimate the characteristic transmission rate of SARS-CoV-2. However, despite its flexibility and mathematical elegance, the model introduces some important limitations. To determine whether new variants of the SARS-CoV-2 are more transmissible than their predecessors, data analysis must cover the entire pandemic outbreak and include the effects of preventive measures and contagion policies taken by populations and governments, respectively. The original SIR model does not account for the social preventive response which characterises the new normal, e.g., social distancing, mask wearing, limited commuting, remote working, or local curfews and lockdowns, to name but a few examples. Fitting data to a model which does not capture how these important social changes affect the spreading of the virus is not a reliable method to discern the transmission (β), recovery (α) and basic reproductive ($R_{0}=\frac{\beta }{\alpha }$) rates. For instance, different values of $R_{0}$ for the same virus with the same inherent properties under different social contexts, e.g., partial and full lockdown, are obtained. Data for the United Kingdom suggest much higher values for β and $R_{0}$ during September–December 2020 if analyzed under the SIR premises [2]. Despite the good fitting of data achieved over limited temporal windows [2,3,4,5], there are two important limitations compromising the accuracy of the predictions: (a) one cannot fit the whole temporal series, characterized by multiple infection waves, and indeed the fit would eventually diverge; and (b) the SIR model, or the equivalent logistic growth model [2], would never forecast a second or further upsurge in cases. Refined SIR models to include additional factors, such as “shield immunity” [6], do not come to our rescue. Indeed, despite their increased flexibility, they are still only capable of showing a single wave. Additionally, many studies fitted SIR-like models to data from the last stages of the first wave—even though, as highlighted above, the models suffer from the inherent limitation of a single-wave prediction—thus effectively assuming that the epidemic was coming to an end. Yet, it was already known at the time that the number of cases was decreasing because of the preventive measures, which in turn, should have been sufficient to abandon the corresponding models. Thus, current studies are incomplete and an alternative approach is necessary.

The main results of our study are as follows. Consideration of the full-history of the data with a controlled SIR model (Figure 1b) avoids the drawbacks of previous models, by capturing the essence of how the new normal affects the population of infected people. This unveils unique and constant β and $R_{0}$ for the entire pandemic. Thousands of mutations have emerged in the SARS-CoV-2 genome since the first outbreak in 2019, and only the UK strain, known as B.1.1.7, is being reported as a more “aggressive” form of the virus, because of an alarming surge in new cases thought to be correlated with the new UK variant, and was one of the reasons for the lockdown imposed in the UK at the beginning of 2021, e.g., Ref. [7]. According to the law of parsimony: chose the simplest explanation from those that fit. Indeed, our results show that the fierce increase in cases over December 2020–January 2021 is captured without the need for a more transmissible variant, suggesting that genomic data during the pandemic might have been overinterpreted. Our approach includes characteristic parameters which could be pivotal in the decision-making process in the coming months. For instance, there seems to be an inertia of society which plays a crucial role in the flattening of the curve. For preventive measures to be effective, these should be encouraged quite early in the surge of cases, taking into consideration the inherent social inertia, which typically leads up to a three-week delay until society gets to its maximum level of alert. We also include the effect of vaccination, and show that social relaxation as of March 2021 without fulfilling a sufficient vaccination rate (determined below) will potentially lead to a new wave of infections over May–June 2021, independently of the more strict lockdown imposed since January 2021 (relaxed on April 2021).

## 2. Methods

### 2.1. Population Dynamics

The total population is split into four groups: susceptible (*S*), infected (*I*), recovered (*R*) and vaccinated (*V*), as illustrated in Figure 1. This last category is essentially the same as the recovered one, but with the particularity that vaccinated subjects do not need to get infected to recover, and they are immediately removed from the susceptible group. For the sake of simplicity, it is assumed that the vaccination is 100% effective. The groups follow the delayed dynamical system:

(1) $$\begin{array}{cc} \frac{dS}{dt}= & \;-\frac{SI}{N}\left(1-A\left(t-\tau \right)\right)\beta -\Theta \left(t-t_{\nu }\right)\;\nu N\\ \frac{dI}{dt}= & \;\frac{SI}{N}\left(1-A\left(t-\tau \right)\right)\beta -\alpha I\\ \frac{dR}{dt}= & \;\alpha I\\ \frac{dV}{dt}= & \;\Theta (t-t_{\nu })\;\nu N \end{array}$$

where $R+V=\int_{0}^{t}ds\;\left(\alpha I\left(s\right)+\nu N\right)$, N=S+I+R+V is the total population, which in this study we have approximated to 60 million people for simplicity. The parameters β,α and ν are the transmission, recovery, and vaccination rates, respectively, Θ is the Heaviside step function and $t_{\nu }$ the time when the vaccination campaign starts. The function A(t−τ) represents the percentage of susceptible individuals using effective preventive measures at time t−τ. Here, τ is a characteristic time for preventive measures to become apparent, which is assumed to be 14 days, also referred to as the incubation period. Ref. [8] The functional form for the awareness function is postulated to be:

$$A\left(t\right)=\sum_{k}\frac{\eta^{k}}{2}\left\{\left[1+\mathrm{erf}\left(\frac{t-t_{0}^{k}}{\delta_{i}}\right)\right]-\left(1+\mathrm{erf}\left(\frac{t-(t_{0}^{k}+T^{k})}{\delta_{r}}\right)\right)\right\},$$

where $\eta^{k}\in \left[0,1\right]$ is the effectiveness of the preventive measures taken in the k-th wave, $T^{k}$ is related to the time extension of these measures, and $\delta_{i,r}$ are the social inertia (*i*) and relaxation (*r*) time scales, respectively.

Equation (1) recovers the free SIR model when A=0 and ν=0, as can be readily checked. For A≠0 we get a controlled SIR model. The initial condition we used in this study: $I_{0}=1$ (number of infective cases reported on 11 January 2020), $S_{0}=N-I_{0}$ and $R_{0}=V_{0}=0$. Thus, fitting of five parameters is needed, and this is done with data of the first wave only. The fitting procedure is further explained in what follows.

### 2.2. Training and Testing the Model

The full dataset, *Y*, was re-normalised to $\tilde{Y}$, by using a linear fit, z=b+mx, to the number of daily CoVid-19 tests per thousand individuals given in Ref. [9], with b=0 and $m=0.0191\;\mathrm{test}/10^{3}\mathrm{people}/d$ (from 0 to 7 $\mathrm{test}/10^{3}\mathrm{people}$ in 366 days), so that $\tilde{Y}=\left(1+\left(\frac{1}{z}-\frac{1}{z_{\mathrm{last}}}\right)\right)Y$. We then use non-linear least squares to fit the daily new cases $\Delta =\frac{d(I+R)}{dt}$ to the first-wave data (training dataset). For the free model the fitting yields: $\beta =0.515d^{-1}$ and $\alpha =0.420d^{-1}$ so that $R_{0}=\frac{\beta }{\alpha }≃1.23$. For the controlled model we get: $\beta =0.211\;d^{-1}$, $\alpha =0.102\;d^{-1}$, η=0.65, 140d, $\delta_{a}=21\;d$ and $\delta_{r}=45\;d$, which yields $R_{0}≃2.068$. To test the models, we numerically integrate (1) for both cases, i.e., A(t)=0 and A(t)≠0. The controlled-model prediction for new infections grows exponentially as of September 2020 (testing dataset) when the first wave of preventive measures would vanish according to the summer trend. With $\delta_{i,r}$ fixed from the first wave, we fit the parameters η and *T* of a second social response to unveil the behavioural changes adopted against the apparent second wave of cases, obtaining a maximum of social response by mid–end October 2020. This is in perfect agreement with the declaration of the UK Prime Minister “seeing a second wave” on 18 September 2020 [10], and his statement on coronavirus where the three-tier restrictions system was imposed on 12 October 2020 [11]. For predictions as of January 2021, we introduce a third wave of measures aiming at an 70% of effectiveness, i.e., η=0.70, starting in January and ending in April 2021, T=90d, which represents the current contagion policies being taken by the UK government. Finally, we adopt three potential values for ν: $\nu_{1}=0.1\%\;d^{-1}\left(∼\frac{75\times 10^{3}}{N}\;d^{-1}\right)$, $\nu_{2}=0.2\%d^{-1}\;\left(∼\frac{120\times 10^{3}}{N}\;d^{-1}\right)$ and $\nu_{3}=0.4\%\;d^{-1}\;\left(∼\frac{200\times 10^{3}}{N}\;d^{-1}\right)$, with $\nu_{3}$ being the current vaccination target since January 2021 [12].

## 3. Results and Discussion

**Figure 2:**
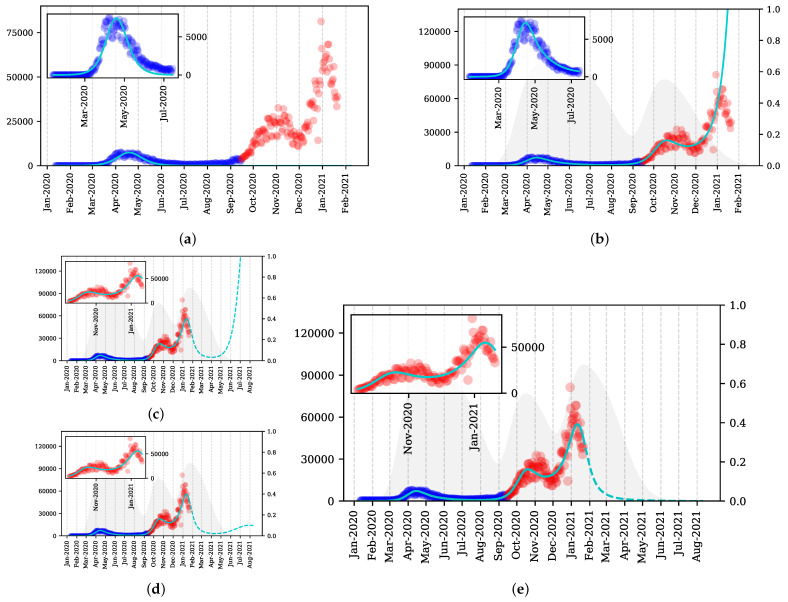
SARS-CoV-2 new daily cases (left axis): blue circles for first-wave data used to fit free and controlled SIR models (light-blue lines), red circles for second- and third-wave data used to test the models and their predictions (light-blue dashed lines). (**a**) Free SIR model captures the essence of the time evolution of new CoVid-19 cases over March–July 2020 (inset plot), but totally fails to predict the second and third waves. (**b**) The controlled SIR model without vaccination fits better to first-wave data (inset plot) than the free version. The grey area represents the effectiveness of preventive measures, A(t) (right axis). The first wave of social awareness is fit together with β and α, showing a maximum of effectiveness $\eta_{1}≃65\%$, social inertia $\delta_{i}≃21\;d$, and social relaxation starting at mid June 2020, with prediction of no measures in $\delta_{r}≃45\;d$ after relaxation begins. The second wave of social awareness begins in September (confirmed by the Prime Minister [10]), reaching $\eta_{2}≃60\%$ by mid October 2020 (three-tier system was introduced [11]). The upsurge of CoVid-19 cases in December 2020 is again a consequence of social relaxation. (**c**–**e**) Controlled SIR model with vaccination rates: $0.1\%d^{-1},\;0.2\%d^{-1},$ and $0.4\%d^{-1}$, respectively, along with a third-wave of preventive measures (expected to reach maximum effectiveness, $\eta_{3}=70\%$, by the mid January 2021). To avoid a fourth wave, the vaccination campaign would need to deliver $∼200\times 10^{3}$ vaccines per day (∼0.4%N/d) as of the first week of January 2021.

Figure 2 reports curve fits and predictions of the free and controlled SIR models. The free version (Figure 1a) fits well the data of the first wave of infections from March to June 2020, but completely fails to predict any second or further wave, as can be seen in Figure 2a. This is because, in a free SIR model, the decay of the infected cases is only possible when the pandemic is already in recession. (As we know now and back in June 2020, this was not the reason for the decrease in cases at the time; the reduction of susceptible people is rather due to preventive measures.) Yet, it has been a standard way of extracting estimates for β,α and $R_{0}$. Some works even published estimates for these quantities via manual fitting to data [5], effectively a trial-and-error approach. The authors justified this by asserting that a rigorous non-lineal fitting did not follow the data as close as their approach. However, instead of imposing the SIR model and changing the fitting method to achieve agreement with the model, the disagreement with a nonlinear fit simply means that the model should have been abandoned altogether.

Not only does the controlled SIR model fits better the first-wave data (inset plot, Figure 2b), but it also captures the reason for the decrease in cases from mid-April to August 2020, namely a wave of social awareness (A) which effectively reduced the number of susceptible individuals. A embodies both contagion policies and the citizens’ efforts made to “flatten the curve”, e.g., wearing masks, reducing traveling or self-isolating. Moreover, the model predicts a sudden rise in cases when society relaxes, because the downtrend in new infections is not related to the end of the pandemic but with the temporary removal of susceptible candidates from the system. This is precisely what happened from July to September 2020, and what eventually led to the surge in cases in early September 2020. This sharp increase immediately raised the alarm [10,13], and A started growing again, reaching maximum effectiveness when the three-tier restrictions system was imposed [11]. However, these measures were not sufficient to flatten the curve and a new increase appeared in December 2020 because of a gradual relaxation over the month of November 2020. By incorporating new waves of preventive measures in A the model reproduces the above observations, as illustrated in Figure 2b–e. This provides evidence of the predictive capabilities of the model.

Figure 2c–e reveal the effects of the third lockdown imposed on 4 January 2021 and of the vaccination campaign with rates $0.1\%d^{-1},\;0.2\%d^{-1},$ and $0.4\%d^{-1}$, respectively. The trends illustrate that unless the campaign delivers $∼200\times 10^{3}$ vaccines per day, a fourth wave is unavoidable. At the same time, if the vaccination rate is less than $100\times 10^{3}$ vaccines per day, the fourth wave will be as severe as previous ones.

**Figure 3:**
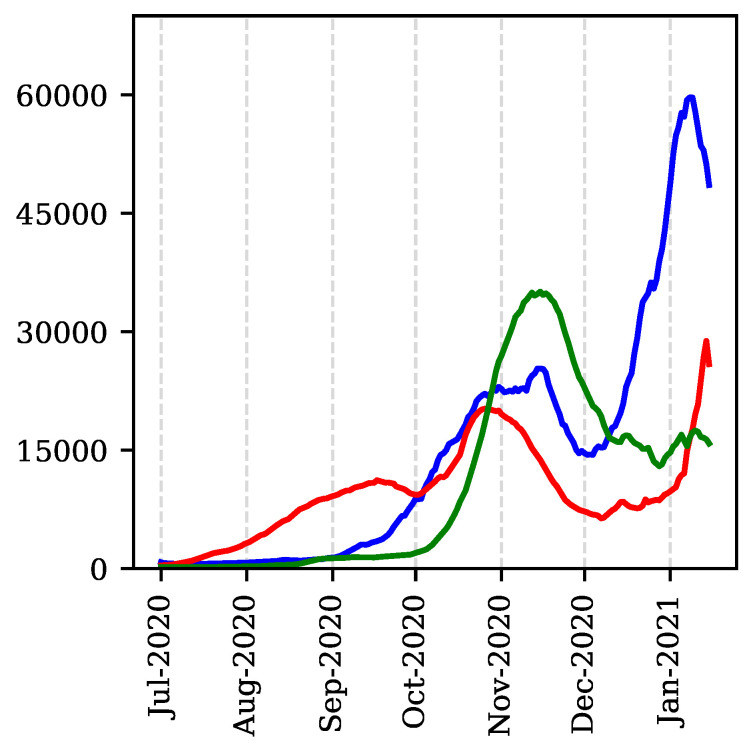
Daily new cases (7-day moving average): UK (blue), Spain (red) and Italy (green).

Analysis of European countries’ data depicted in Figure 3, including countries severely hit by the pandemic, like Spain and Italy, reveals that virtually in all cases, the UK pattern persists and similar conclusions can be drawn. This means, social relaxation—typically two-to-three months—driving an abrupt increase of cases, followed by increased awareness and preventive measures leading to temporal stagnation, which is then followed by further growth, i.e., a third, more powerful, wave.

## 4. Conclusions

We have put forward an extended version of the classical SIR model for infectious diseases, a controlled SIR model, with the aim of modeling and forecasting the evolution of the SARS-CoV-2 infections experienced in the UK. Our model incorporates two new terms that capture the effects of social awareness and vaccination. The results of fitting our model to real data for the UK shows that social awareness and preventive measures temporarily reduce the infectious rate, but as soon as society becomes relaxed, the number of infections raises dramatically. This is, e.g., what we observed by mid-September 2020, which unfortunately coincided with the appearance of a new variant of the virus, first detected in the UK. This unfortunate coincidence, and the fact that the new variant was more stable than the previous ones, led to conclude that the UK variant was much more infectious. Our results show that a unique infection rate (hence, same infectious rate for all the coexisting variants) is compatible with the waves of infections that have been observed in 2020. Indeed, with such a unique infection rate, the model not only predicts correctly the second peak of infections which took place between mid September 2020 and December 2020, but also forecasts the peak of the wave in January 2021. It also enables us to scrutinize different scenarios depending on the rate of vaccination as of January 2021. Finally, there are a number of interesting extensions of the model developed here, for instance to account for new susceptible categories associated with different infection rates according to the age distribution of the population. Another related problem is coupling the equations with a model of human mobility incorporating the effects of transportation. We shall examine these and related questions in future studies.   

## Data Availability

All data for the analysis was collected from https://www.worldometers.info/coronavirus/country/uk/, accessed on 28 April 2021. The code used in the creation of this manuscript is available at https://github.com/migduroli/covid-uk-variant/, accessed on 28 April 2021. Model simulations were numerically integrated using odeint from scipy [14], a Python wrapper for LSODA from the FORTRAN library ODEPACK [15]. The non-linear square fittings where carried out by using curve_fit from scipy [14].
